# *In situ* metabolism in halite endolithic microbial communities of the hyperarid Atacama Desert

**DOI:** 10.3389/fmicb.2015.01035

**Published:** 2015-10-06

**Authors:** Alfonso F. Davila, Ian Hawes, Jonathan G. Araya, Diego R. Gelsinger, Jocelyne DiRuggiero, Carmen Ascaso, Anne Osano, Jacek Wierzchos

**Affiliations:** ^1^Carl Sagan Center, SETI, Mountain View, CA, USA; ^2^Gateway Antarctica, University of Canterbury, Christchurch, New Zealand; ^3^Laboratorio de Microorganismos Extremófilos, Instituto Antofagasta, Universidad de Antofagasta, Antofagasta, Chile; ^4^Department of Biology, Johns Hopkins University, Baltimore, MD, USA; ^5^Grupo de Ecología y Geomicrobiología del Sustrato Lítico, Departamento de Biogeoquímica y Ecología Microbiana, Museo Nacional de Ciencias Naturales (MNCN), Consejo Superior de Investigaciones Científicas (CSIC), Madrid, Spain; ^6^Department of Natural Sciences, Bowie State University, Bowie, MD, USA

**Keywords:** Atacama, halite, deliquescence, metabolism, endoliths

## Abstract

The Atacama Desert of northern Chile is one of the driest regions on Earth, with areas that exclude plants and where soils have extremely low microbial biomass. However, in the driest parts of the desert there are microorganisms that colonize the interior of halite nodules in fossil continental evaporites, where they are sustained by condensation of atmospheric water triggered by the salt substrate. Using a combination of *in situ* observations of variable chlorophyll fluorescence and controlled laboratory experiments, we show that this endolithic community is capable of carbon fixation both through oxygenic photosynthesis and potentially ammonia oxidation. We also present evidence that photosynthetic activity is finely tuned to moisture availability and solar insolation and can be sustained for days, and perhaps longer, after a wetting event. This is the first demonstration of *in situ* active metabolism in the hyperarid core of the Atacama Desert, and it provides the basis for proposing a self-contained, endolithic community that relies exclusively on non-rainfall sources of water. Our results contribute to an increasing body of evidence that even in hyperarid environments active metabolism, adaptation, and growth can occur in highly specialized microhabitats.

## Introduction

The Atacama Desert of northern Chile is one of the oldest continuously dry regions on Earth (Clarke, 2006). The long-term mean annual precipitation in the driest parts of the desert, the so-called hyperarid core, is less than 1 mm, although consecutive rainfall events can be interspaced a decade or longer (Rundel et al., 1991; McKay et al., 2003). This extreme and prolonged dryness is manifested in the ecology of the region, which is devoid of plants and animals, while soils contain a meager population of microbial cells at an average cell concentration of 10^3^–10^5^ cells/g soil (Connon et al., 2007; Crits-Christoph et al., 2013). Carbon cycling in these soils occurs in timescales of thousands of years (Ewing et al., 2008), comparable to rates measured in Antarctic permafrost and in deep subsurface sediments (Palmer and Friedmann, 1990; Phelps et al., 1994; Lin et al., 2006). Survival strategies commonly used by poikilohydric microorganisms in hot and cold deserts, such as the colonization of the ventral side of translucent rocks, fail in the driest parts of the Atacama (Warren-Rhodes et al., 2006).

In stark contrast to the biologically lean soils and barren lithic substrates, an abundant and diverse community of microorganisms can be found in the fossil continental evaporites or salars that are scattered in the hyperarid central valley (Wierzchos et al., 2006), and have been hydrologically inactive since the Miocene (Pueyo et al., 2002). The endolithic community is found inside halite (NaCl) nodules on the surface of the salars, and comprises a single species of cyanobacteria, common to all salars so far investigated, along with diverse heterotrophic bacteria and archaea, and occasionally algae (de Los Ríos et al., 2010; Robinson et al., 2015). The carbon isotopic composition of the lipid fraction suggests that carbon cycling in the halite nodules is ongoing, with carbon turnover times of years to decades depending on water availability (Ziolkowski et al., 2013).

Deliquescence-activation of variable chlorophyll fluorescence in the halite cyanobacteria has been shown to occur in controlled laboratory conditions using pulse amplitude modulated (PAM) fluorometery (Davila et al., 2013). In this context, the PAM technique exploits properties of photosystems that occur only with active electron transport, and it provides a useful indicator of their status during wetting/drying cycles (Lange et al., 2001; Wieners et al., 2012). However, variable chlorophyll fluorescence (quantum yield) does not indicate the activity of carbon-fixation pathways. As of yet, there is no direct evidence that the halite endolithic communities are capable of fixing carbon under the salt-saturated conditions that prevail in liquid films that result from deliquescence or capillary condensation.

Here, we used *in situ* PAM fluorometry to analyze the response of the photosystem II [PS(II)] in halite endolithic cyanobacteria to natural fluctuations of temperature and relative humidity (RH), and demonstrate that these microorganisms are metabolically active under natural conditions. We complemented these measurements with controlled laboratory experiments to determine whether microorganisms isolated from halite nodules were capable of light-driven carbon fixation and light-induced oxygen evolution. We argue that demonstration of these two properties—active fluorescence *in situ* when brine films are present and the ability to photosynthesize and respire in saturated brines—constitute evidence to support the metabolically active status of halite endolithic communities.

## Materials and Methods

### Field Site, Sample Collection, and Sensor Deployment

**FIGURE 1:**
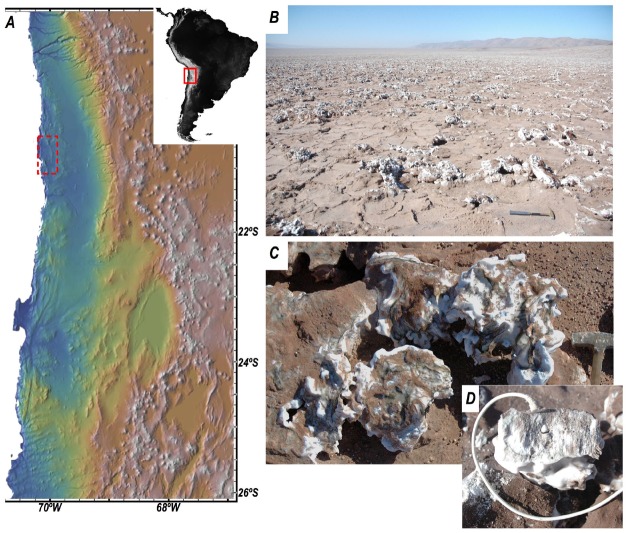
**(A)** Elevation map showing the location of Salar Grande (red dotted rectangle) in the Atacama Desert of northern Chile. **(B)** Panoramic view of Salar Grande (looking south) with salt polygons and nodules in the foreground. **(C)** Close-up view of a colonized salt nodule. The surface of the nodule shows the typical dark-green colorization due to exposed endolithic communities. **(D)** T/RH sensors inside a salt nodule (image taken at the end of the field experiment).

Field investigations were conducted during 2013 and 2014 in Salar Grande (S20°57′10′/W70°01′07′), a fossil salar located near the coastal cordillera, in Northern Atacama. The present-day dry, fossil salar consists of a massive salt body approximately 70–90 m thick, with thick beds of almost pure halite formed approximately 1.8–5.3 million years ago, when the basin became hydrologically inactive. The surface of the salar is shaped in the form of polygons and nodular structures characteristic of this type of evaporitic settings (Figures 1A–C; Artieda et al., 2015). A Relative humidity/temperature (RH/T) sensor (HOBO^®^ S-THB-M002) was inserted inside a salt nodule within the colonized zone (Figure 1D) and set to record data every 10 min. At the same time, we monitored the activity of the PSII in endolithic communities over four consecutive days (see below) during September 2014. Whole colonized halite nodules were also collected in sterile plastic bags for laboratory investigations.

### Pulse Amplitude Modulated Chlorophyll Fluorescence

Photosystem II activity was measured using pulse amplitude modulated (PAM) Chlorophyll fluorescence to monitor changes in the fluorescence yield of chlorophyll-a in response to natural daily cycles. PAM fluorescence is a non-invasive technique that allows measuring transient, low levels of photosynthetic activity in the endolithic community. Unlike methods based around oxygen or carbon dioxide dynamics, it is unaffected by liquid–solid phase shifts and thus not affected by the degree of hydration of the substrate and changes in gas solubility (Davila et al., 2013). Variable fluorescence is estimated by first measuring fluorescence under ambient conditions (*F*), then measuring maximum fluorescence during application of short (0.6 s) saturating light pulse (*F_m_*′). Variable fluorescence is calculated as (*F_m_*′ – *F*)/*F_m_*′ and when measured under ambient irradiance is referred to as an estimate of the yield of PSII or Y_II_. While fluorescence is a property of chlorophyll regardless of physiological state, variable fluorescence requires *F_m_*′ ≠ *F_o_* and is evident only when PSII electron transport chains are active. For a detailed description of the saturation pulse method, see Schreiber (2004); for its use in determining the onset of photosynthetic activity on rewetting of dry lichens, see Lange et al. (2001).

We used a Walz Water PAM fluorometer (Walz Mess- und Regeltechnik, Germany) with an optical fiber sensor to quantify the minimum (*F*) and maximum (*F_m_*′) fluorescence yields of freshly exposed endolithic communities from the inside of halite nodules. Surfaces were exposed by cracking open nodules using a geological hammer, under shaded conditions. Samples were then transferred immediately to a near-dark box, the measuring optical fiber was placed against visibly colonized parts of the halite nodule, and fluorescence parameters determined as quickly as possible. While PAM fluorescence is a non-invasive technique, exposing the endolithic colonies for measurement implies that the same region cannot be analyzed at different time points to monitor changes in response to daily cycles, since conditions would no longer be ambient. The alternative is to analyze different samples under the assumption that the physiological state of the endolithic colonies from different nodules is comparable at the same time of the day. We therefore established a protocol whereby freshly exposed interior surfaces were measured at time intervals of 3 h between dawn and dusk. At each measuring time we analyzed three different nodules. Each nodule was broken down into several fragments (*N* = 3–4), and for each fragment we collected 3–5 fluorescence readings within the colonized region.

### Metabolic Activity Assays

Endolithic cells were extracted from halite nodules in the laboratory by dissolving colonized portions of nodule in sterile water. The goal was to create a cell suspension in saturated brine, estimated at 360 g L^–1^ at 25°C, and to this end approximately 100 *g* of colonized nodule was added to sterile water into which 25 g of non-colonized halite was already dissolved to minimize osmotic shock. After the salt/water mix was equilibrated, a layer of undissolved salt remained, and the liquid brine phase was visibly green-tinted. The suspension of cells was dispensed into 25, 10 ml glass tubes each with a rubber septum seal, taking care to avoid air bubbles.

#### Carbon Fixation

Fifteen tubes were randomly selected for carbon uptake incubations and injected with 0.2 ml of ^14^C labeled bicarbonate (final concentration 3.33 μCi ml^–1^), allowing the displaced suspension to leave the tube via a second needle. Of these, five were immediately filtered through a 0.2 μm diameter Whatman GF/F filter, as time 0 controls. These filters were placed into scintillation vials and 0.5 ml of 10% hydrochloric acid added to halt metabolism and to drive off remaining labeled inorganic carbon and frozen. Of the remaining 10 tubes, five were covered in aluminum foil to act as dark controls, and all tubes were placed in an illuminated incubator (∼10–20 μmol photons m^–2^ s^–1^ on a 16:8 light:dark cycle at 20°C) for 72 h. After 72 h incubation the entire contents of each tube were filtered and preserved as described above. Radioactive counts per minute (cpm) were determined by liquid scintillation spectrometry using a Packard Tri-Carb 2200CA Scintillation Counter (Waltham, MA, USA).

Carbon uptake was also measured in partially ground halite nodules that exhibited notable zones of colonization. 1.5 g aliquots of ground halite were dispensed into 30 ml serum bottles with 2 ml of 20% NaCl. These conditions maintained most of the ground halite in solid form, with an atmosphere at ∼75% RH, the air equilibrium value over a saturated sodium chloride solution. Serum bottles were incubated with 2.5 μCi of ^14^C labeled bicarbonate (specific activity 40–60 mCi/mmol; Perkin Elmer, MA, USA) under light or dark conditions for 5 days. A potential redox electron donor, ammonium chloride, was added to a subset of samples at a final concentration of 1 mM. Each experiment was done in triplicate. Samples were processed as described above to determine radioactive counts.

#### Oxygen Exchange

Ten glass tubes were prepared as described above for measurement of oxygen metabolism. Dissolved oxygen was measured at time 0 using a PreSens TX3 oxygen meter connected to a PreSens needle-mounted optical oxygen micro-sensor (PreSens GmbH, Regensburg, Germany, http://www.presens.de). The needle sensor was injected into the tube through the septum and dissolved oxygen concentration determined after a 20 s equilibration time. Five tubes were then covered in aluminum foil for dark incubation, and all 10 were placed in the incubator as described above. After 24 and 48 h the oxygen concentration in each tube was re-measured.

## Results

### Environmental Data

**FIGURE 2:**
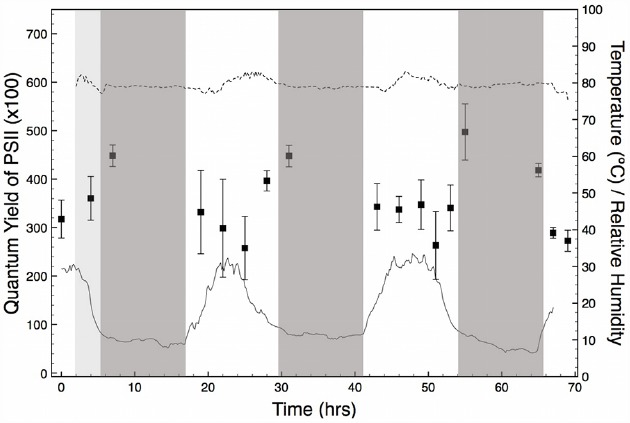
**Quantum yield of PS(II) measured in freshly exposed interiors of halite nodules for a period of 4 days and three nights (black squares).** Nodules were measured at time intervals of 3 h between dawn and dusk. Each data point is the average of 25–40 measurements obtained from three different nodules. Error bars represent standard deviations. The solid line (T) and dotted line (RH) show the conditions inside a reference nodule measured at time intervals of 10 min for the duration of the experiment. Night periods are indicated as dark-gray. An overcast period with fog at the beginning of the experiment is indicated in light gray.

Throughout the period of PS(II) activity monitoring (4 days, and three nights) there was no precipitation and skies remained mostly clear. On the first day, a period (c.a. 5–10 h) of overcast delivered fog to the salar, but this did not recur during the experiment. The RH/T data measured inside a halite nodule showed a daily cycle of temperature from approximately 8 to 32°C that was coincident with the solar cycle. In contrast, RH inside the nodule was constant at approximately 80% throughout the entire period of field observations (Figure 2). The constant, high value of RH inside the nodule corresponds to the equilibrium RH of a saturated solution of NaCl. As explained in detailed in previous studies (Davila et al., 2008, 2010; Wierzchos et al., 2012), this high and constant RH value is indicative of the presence of liquid water inside the halite nodule.

### Pulse Amplitude Modulated Chlorophyll Fluorescence

Measurements of variable chlorophyll fluorescence showed that cyanobacteria’s PS(II) were potentially active during the entire period of observation (Figure 2). Throughout the experiment, we found that fluorescence was localized to the green-stained parts of the nodule interiors that contain the cyanobacteria. *Y_II_* values in these areas were comparable between nodules analyzed at the same time of the day. This finding validated our initial assumption that the physiological state of the endolithic colonies from different nodules was comparable, justifying our experimental approach. *Y_II_* activity was cyclic over a 24 h period, with maximum values observed shortly after sunset and before sunrise. While significant variable fluorescence was still observed during the day, *Y_II_* declined by approximately 20 to 40% and reached a minimum value between 12 pm and 4 pm local time, when temperature and solar insolation were highest (Figure 2). Within this time interval, values of variable fluorescence inside each nodule also showed a larger scattering than at dawn and dusk.

### Metabolic Activity Assays

#### Carbon Fixation

**FIGURE 3:**
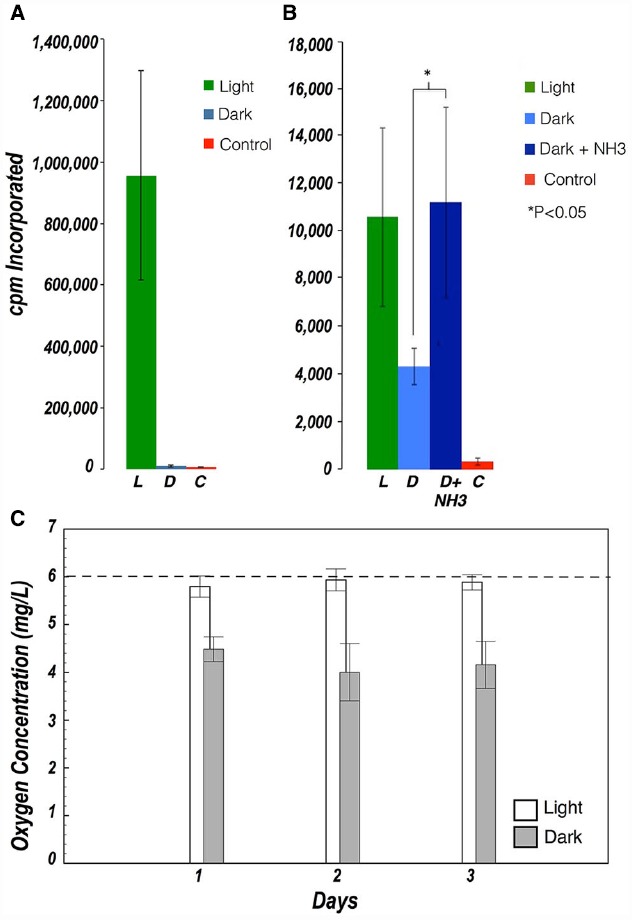
**(A)** Carbon fixation in halite cell suspensions in the presence of light (green) and in the dark (blue) **(B)** Carbon fixation in ground halite in the presence of light (green), in the dark (light blue), and in the dark after addition of NH_3_ as an electron donor (dark blue). Controls with no viable cells are shown in red. Dark Carbon uptake was only observed in ground samples (not in cell suspensions) and was stimulated after addition of NH_3_. Values represent the average of 3–5 measurements. Error bars represent standard deviations. **(C)** Evolution of oxygen concentration in cell suspensions exposed to light (white) and dark (gray). The dashed line shows the oxygen concentration measured at the beginning of the experiment.

Incubations with radiolabeled inorganic carbon were deliberately long as the rate of photosynthesis was expected to be low. Absolute rates of carbon fixation could not be determined because the concentration of inorganic carbon in the brine, and the rate of depletion thereof during these long incubations, could not be measured. Thus all rates are presented as cpm collected over the incubation period. Significant uptake of radiolabeled carbon was observed in cell suspensions and in ground halite samples exposed to light, indicating that photosynthesis was the main pathway for carbon fixation. Significant carbon fixation was also observed in the dark in ground halite samples, albeit to a lesser extent than in the light. Dark fixation in the ground halite was stimulated by addition of NH_3_ as an electron donor, but it was not observed in the incubation with cell suspensions (Figures 3A,B).

#### Oxygen Exchange

Evidence of metabolic activity in the cell suspensions was further supported by oxygen concentration measurements during light/dark incubations over the course of 48 h. In the dark, oxygen concentration decreased significantly from those in the light, indicating respiratory consumption in all samples, but which was partially offset by photosynthetic oxygen production in samples exposed to light (Figure 3C).

## Discussion

Liquid water is a fundamental requirement for life, and yet there are specialized organisms adapted to survive, and thrive, under severe water deprivation (cf. Pointing and Belnap, 2012). Until recently, the driest parts of the Atacama Desert were considered an exception, based on the virtual absence of hypolithic microorganisms and biological soil crust frequently found in deserts (Warren-Rhodes et al., 2006; Pointing and Belnap, 2012). However, the discovery of an abundant and diverse endolithic community inside halite nodules in ancient fossil salars (Wierzchos et al., 2006) suggested that metabolic processes might be operating in this extremely dry region within specialized micro-niches.

Key metabolic processes for maintenance of an endolithic microbial community include those that provide a source of organic carbon. Using *in situ* PAM fluorometry, we showed that PS(II) of cyanobacteria inside halite nodules were active for periods of at least several days during and after wetting events. This data, together with the measurement of carbon fixation in the presence of light and the difference in oxygen concentration under dark/light conditions, demonstrate that photosynthetic cyanobacteria inside the nodules are a source of organic carbon to the whole endolithic community, which also includes heterotrophic bacteria and archaea (de Los Ríos et al., 2010; Robinson et al., 2015). The significant increase in net carbon fixation in the dark, when an electron donor (ammonia) was added to the ground halite, suggests that carbon fixation might also be mediated by chemolithoautotrophic microorganisms present in the endolithic community. A preliminary metagenomic survey (data not shown) indicated the presence of ammonia oxidizing bacteria in the halite community but additional studies are needed to conclusively link taxonomy with function.

Carbon fixation in the dark was seemingly abolished in cell suspensions when compared to ground halite, suggesting that the structure of the endolithic microbial cell aggregates played a significant role in this process. This might be the result of a collapse of the habitat, disruption of the gas exchange interface, displacement of micro-colonies, or increased potential for osmotic shock. However, we cannot rule out that the apparent dark carbon fixation in ground halite may be the result of an anaplerotic reaction via the Wood-Werkman pathway, where CO_2_ is incorporated into succinate through a pyruvate carboxylase reaction (Nielsen, 1960). This process can be enhanced by ammonia in N-limiting conditions (Morris et al., 1971), explaining the increase carbon fixation we observed in the ammonia-supplemented samples. Further studies are required to fully elucidate the dynamic processes that occur in the halite environment. Nevertheless, our findings suggest that the metabolic diversity in the halite is much broader than previously expected and that the structure of the halite likely provides niche environments within the community itself.

The major ecological advantage of the salt substrate is its capacity to condense liquid water directly from the vapor phase, and to retain brines for extended periods of time after a wetting event, even under the extremely dry conditions reported for the Yungay area (Davila et al., 2008; Wierzchos et al., 2012). This explains the buffering of RH at a continuous 80% inside the halite of the Salar Grande area, which is at or close to the deliquescence point of halite. The active role of the salt substrate in delivering water to the community sets it aside from other substrates from the Atacama Desert where endolithic communities have also been found. In these substrates, which include gypsum (Dong et al., 2007; Wierzchos et al., 2011), calcite and rhyolite (DiRuggiero et al., 2013), and ignimbrite (Wierzchos et al., 2013), microorganisms have to rely on scarce atmospheric precipitations such as rainfall, fog or dew. Water retention after a moist event is another key advantage of lithic habitats. Here too the halite substrate is first among equals, as it can retain water in its interior even after weeks of continuous dryness (Davila et al., 2008; Wierzchos et al., 2012), greatly expanding the window of metabolic activity for the endolithic community. This is especially relevant for phototrophs, which in addition to liquid water also require sufficient light as an energy source. Photosynthetic activity in biological soil crusts and lithic substrates in arid desert environments is typically confined to a few hours in the morning, before water accumulated during the night evaporates (e.g., Noy-Meir, 1973). This limits the capacity of the entire community to sustain a net positive carbon balance, unless respiration at night is balanced by carbon fixation via photosynthesis during the day. In the halite nodules, our field measurements showed that the cyanobacteria can be active for several consecutive days, and our laboratory experiments revealed potential chemoautotrophy at night, providing several means to ensure a long-term positive carbon balance in the community.

Yet, the salt endoliths must still face challenges. Liquid water in the interior of the nodules is likely to form a saturated brine with a water activity of approximately 0.75 (Davila et al., 2008). Translation of active photosystems to carbon accrual requires the ability within the endolithic community to fix carbon and respire oxygen under this very low water activity. Our previous work revealed a microbial community adapted to this extreme environmental niche, dominated by haloarchaea and with a unique cyanobacteria of the genus *Halothece* (de Los Ríos et al., 2010; Robinson et al., 2015). Solar radiation levels in the Atacama Desert rank amongst the highest in the world, amid the low latitude (c.a. 23°S), elevation (c.a. 1000 m), clear skies, and low atmospheric water content (Cockell et al., 2008). We argue that the halite habitat provides a degree of protection from these extreme environmental stresses and the moisture needed by this singular endolithic microbial community.

Our field measurements suggest that the distribution of endolithic cyanobacteria inside the salt matrix is not random, but the result of a compromise between maximizing light energy inputs and minimizing radiation damage. Evidence for this comes from the cycle of *Y_II_* in the nodules, which showed a reduction under increasingly bright illumination during the day (Figure 2). This reduction represents the photosystems of cyanobacteria light-saturating during the brightest part of the day and returning to their fully open state at night. Analogous patterns have been seen in a range of photosynthetic organisms (e.g., Lange et al., 2001; Hawes et al., 2003). The reduction in *Y_II_* in early afternoon is relatively small (20–30% less than night values) and the rapid recovery on darkening at dusk suggests that these cells are simply reversibly down-regulating photosynthesis. In contrast, persistent photoinhibition or photodamage would cause much greater degrees of reduction in *Y_II_*, and persist for longer periods after removal of the light stress (Hanelt et al., 2006; Takahashi et al., 2010). The absence of persistent photoinhibition is likely due to the distribution of cells below the surface of the nodule, in a “goldilocks zone” where sufficient PAR reaches the cells for net photosynthesis, but where photoinhibition is minimized during peak sun hours. Indeed, incoming solar irradiation is heavily attenuated by the translucent crystal lattice in salt substrates such as halite or gypsum (Oren et al., 1995; Spear et al., 2003). This is tentatively supported by the maximum value of PAR measured within the colonization zone in a representative halite nodule (not shown), which was 0.02% of the incident PAR on the surface of the nodule [maximum values in the Salar Grande typically range between 1900 and 2300 μmol s^–1^m^–2^, Robinson et al. (2015)], and likely insufficient to cause photoinhibition or photodamage to the endolithic community.

Overall, our results confirm for the first time that a diversity of metabolic processes occur, *in situ*, within endolithic microbial communities in the hyperarid core of the Atacama Desert, despite its extreme dryness. Over the long term, development of strategies for carbon fixation and metabolism during periods when deliquescence-based brine is available has allowed the accumulation of relatively high cell abundances, and a diversity of microorganisms capable of relatively fast rates of carbon cycling in the interior of salt nodules (de Los Ríos et al., 2010; Ziolkowski et al., 2013; Robinson et al., 2015). This adds to a growing body of evidence that as conditions become increasingly dry, living processes are restricted to specialized micro-niches. Ultimately, the capability to actively provide liquid water to the community, and to retain water after a moist event, make the halite nodules the ultimate substrates for survival under prolonged and extreme environmental dryness. It has been suggested that a similar survival strategy could have evolved on Mars as conditions became increasingly dry, if life ever evolved on the planet (Davila et al., 2010). Here, in addition to sourcing liquid water and providing shelter against UV, the salt substrate might also act as antifreeze, thereby expanding the range of conditions compatible with metabolic processes both in terms of water deficit and temperature.

## Conclusion

In the driest parts of the Atacama Desert active metabolism can still occur in specialized microhabitats such as the interior of salt nodules, due to the capability of the salt substrate to directly condense liquid water from atmospheric water vapor, and to retain the liquid water after a moist event. This is a unique ecological niche in the sense that the endolithic colonies within each nodule live in isolation, and must therefore form a complete and self-sustainable community. Here, carbon fixation can occur both through oxygenic photosynthesis and potentially ammonia oxidation, pointing to a broad range of metabolic capabilities in the halite community. Within the nodules, photosynthetic microorganisms appear to occupy “goldilocks” habitats favoring slow growth in dim light but minimizing exposure to high solar irradiance, though this balance between avoiding photo-damage while allowing growth may have different solutions in salars with slightly different climates.

### Conflict of Interest Statement

The authors declare that the research was conducted in the absence of any commercial or financial relationships that could be construed as a potential conflict of interest.
